# Hematopoietic Stem Cell Transplantation Activity and Trends at a Pediatric Transplantation Center in Turkey During 1998-2008

**DOI:** 10.5505/tjh.2012.78300

**Published:** 2012-06-15

**Authors:** Volkan Hazar, Gülsün Karasu, Vedat Uygun, Mediha Akcan, Alphan Küpesiz, Akif Yeşilipek

**Affiliations:** 1 Akdeniz University, School of Medicine, Department of Pediatric Hematology, Oncology and BMT Unit, Antalya, Turkey

**Keywords:** Hematopoietic stem cell transplantation, Pediatric, Transplant-related mortality, Turkey

## Abstract

**Objective:** The aim of this study was to document hematopoietic stem cell transplantation (HSCT) activity and trends at our treatment center.

**Material and Methods: **Data collected over a 10-year period were retrospectively analyzed, concentrating primarily on types of HSCT, transplant-related mortality (TRM), stem cell sources, indications for HSCT, and causes of death following HSCT.

**Results:** In total, 222 allogeneic (allo)-HSCT (87.4%) and 32 autologous (auto)-HSCT (12.6%) procedures were performed between 1998 and 2008. Stem cells obtained from unrelated donors were used in 22.6% (50/222) of the allo- HSCTs. Cord blood was the source of hematopoietic stem cells (HSC) in 12.2% of all transplants. The most common indication for allo-HSCT was hemoglobinopathy (43.2%), versus neuroblastoma (53.1%) for auto-HSCT. The TRM rate 1 year post transplantation was 18.3% ± 2.5% for all transplants, but differed according to transplantation type (23.5% ± 7.9% for auto-HSCT and 17.5% ± 2.6% for allo-HSCT). The most common cause of death 1 year post HSCT was infection (35.9%).

**Conclusion:** The TRM rate in the patients that underwent allo-HSCT was similar to that which has been previously reported; however, the TRM rate in the patients that underwent auto-HSCT was higher than previously reported in developed countries. The selection of these patients to be transplanted must be made attentively.

## Abstract (tr)

**Amaç:** Bir pediatrik merkezde yapılan hematopoietik kök hücre nakil (HKHN) aktiviteleri ve yönelimleri hakkında bilgi vermektir.

**Gereç ve Yöntemler:** On yıllık bir zaman dilimindeki veriler geriye dönük olarak değerlendirilmiştir. Çalışmanın sonlanım noktaları olarak, gerçekleştirilen HKHN çeşitleri, nakil ilişkili ölüm hızları, kök hücre kaynakları, nakil endikasyonları ve nakil sonrası ölüm nedenleri olarak belirlenmiştir.

**Bulgular:**1998-2008 zaman diliminde, 222’si (%87.4) allojeneik ve 32’si (%12.6) otolog olmak üzere toplam 254 HKHN gerçekleştirildi. Allojeneik olguların %22.6’sı akraba dışı donör kaynaklıyken, tüm nakillerin %12.2’si kordon kanı ile yapılan nakillerdi. Hemoglobinopatiler, allojeneik HKHN’lerinin en sık endikasyonu iken, otolog olgularda en sık endikasyon, nöroblastomaydı. Tüm grupta nakil ilişkili ölüm hızı %18.3±2.5 olarak saptandı. Nakil ilişkili ölüm hızı 1. yılda, otolog nakillerde %23.5±7.9 ve allojeneik nakillerde %17.5±2.6’dı. HKHN sonrasında ilk 1 yıl içindeki en sık ölüm nedeni, infeksiyonlar olarak belirlendi.

**Sonuç:** Nakil ilişkili ölüm hızları allojeneik grupta literatürle uyumlu bulunurken, otolog grupta yüksek bulundu. Bu hasta grubunun seçimi daha detaylı olarak yapılmalıdır.

## INTRODUCTION

Hematopoietic stem cell transplantation (HSCT) is a well-established treatment for many malignant and nonmalignant diseases [1]. The ability to successfully perform allogeneic (allo)-HSCT depends on acceptable matching between donor and recipient human leukocyte antigen (HLA) systems. Graft versus host disease (GVHD) is the most important consequence of donor and recipient mismatch. Immunosuppressive agents, such as prednisolone, methotrexate, and cyclosporine A, are used for GVHD prophylaxis. The ideal for allo-HSCT is to use a fully HLAmatched sibling donor. Cord blood (CB), in which stem cells are immunologically immature and thus require less rigorous HLA matching, can be used for HSCT when there isn’t an adequately matched donor available. Possible complications of HSCT are infection, GVHD, organ toxicity, reduced growth and fertility, secondary malignancy, and relapse or persistence of the underlying disease. 

To date, there has been no large-scale study conducted in Turkey on HSCT strategies used in children. The aim of the present study was to describe the trends in HSCT activity in pediatric patients at a center in Turkey during the 10-year period of 1998-2008.

## MATERIALS AND METHODS

**Data source **

Data were collected from records of HSCT patients. All transplanted patients between 01 January 1998-31 December 2008 were included. The minimal requirement of requested information for this study was patient age, gender, disease, disease stage, stem cell source, donor type, and outcome. Patient data were updated annually to as late as 31 December 2009, i.e. minimum follow-up was 1 year. Informed consent was provided by the parents/ guardians of each patient in accordance with The Declaration of Helsinki.

**Definitions**

HSCT was defined according to European Group for Blood and Marrow Transplantation (EBMT) criteria [2]. HSCT was considered the infusion of hematopoietic stem cells with the intension to replace the recipient’s pre-transplant hematopoietic system. Delayed engraftment was defined as an absolute neutrophil count less than 0.5 x 109 L–1 at 28 d post HSCT. Graft failure (GF) was defined as the absence of donor-derived hematopoiesis. Re-infusion of allogeneic stem cells for graft failure was considered retransplant. Re-infusion of autologous stem cells for nonengraftment was considered a boost, not a transplant. 

Event-free survival (EFS) was defined as the time from transplantation to the date of the last follow-up during complete remission or the first event. Events were considered resistance to transplantation, relapse or progression of primary disease, or death due to any cause. Overall survival (OS) was defined as the time from transplantation to death or the date of the last follow-up. Transplant-related mortality (TRM) was defined as death due to any cause in the absence of relapse or progression of primary disease, including infections, toxicities, and other non-relapse- or disease progression-related causes of death. An alternative donor was any non-sibling family member or unrelated donor. 

**Statistical analysis **

The mean, median, and standard deviation of numerical variables were calculated. Outcomes measured were EFS and OS, based on Kaplan-Meier estimation, and TRM and relapse rates were based on cumulative incidence curves adjusted for competing risks, as appropriate. Comparisons were made using the log-rank test. Statistical analyses were performed using SPSS v.15.0 (Inc., Chicago, IL, USA).

## RESULTS

**Number and characteristics of HSCTs **

In all, 244 first transplants were performed between 1998 and 2008 (allo-HSCT: n = 212 [86.9%] and autologous (auto)-HSCT: n = 32 [13.1%]). Additionally, 10 retransplants (all allo-HSCTs) were performed during the same time period a median 212 d (range: 54-1023 d) after the first transplantation. Median age of the patients was 82 months (range: 7-272 months) and 60.2% were male (Table 1). 

**Indications for transplantation and donor types **

**Figure 1:**
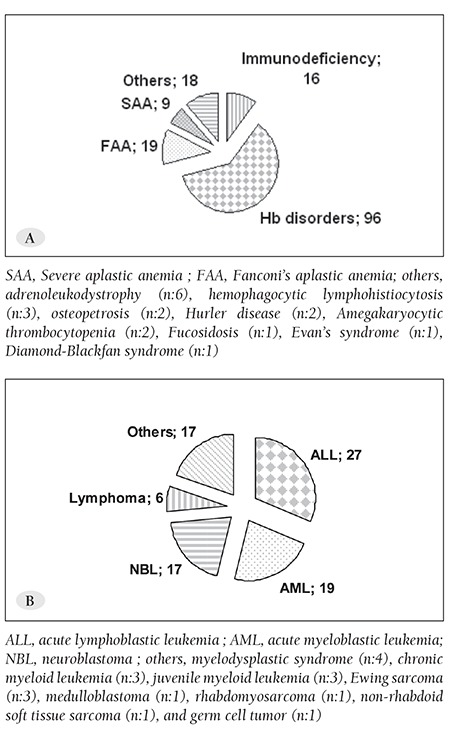
HSCT indications for non-malignant disorders (A)and malignant disorders (B)*

Indications for the first HSCT are shown in Figure 1. The most common malignant disorder indicating HSCT was acute leukemias (n=46 [53.5%]). Most of the patients with a non-malignant disorder had hemoglobinopathy (n = 96 [60.7%]), followed by Fanconi anemia (FA) (n=19, 12.0%). Indications for allo-HSCT (n = 54 [25.5%]) were malignant diseases, including acute lymphoblastic leukemia (ALL) (n=27 [12.7%]), acute myeloblastic leukemia (AML) (n=16 [7.5%]) (3 of the AML patients underwent auto-HSCT), other malignancies (n=11 [5.2%]), and non-malignant disorders (n=158 [74.5%]), including hemoglobinopathy (n = 96 [45.2%]), FA (n = 19 [9.0%]), immunodeficiency (n = 16 [7.5%]), and others (n=27 [12.7%]). Indications for auto-HSCT were neuroblastoma (n=17[53.1%]), followed by lymphomas (n =5[15.6%]). In all, 10 patients underwent re-HSCT (hemoglobinopathy: n = 5; amegakaryocytic thrombocytopenia: n = 1; juvenile myelomonocytic leukemia: n = 1; ALL: n = 1; chronic myelocytic leukemia: n = 1; FA: n = 1). Prior to 2006, the majority of allografts (84.6%) were performed using HLA-matched sibling donors (MSD). Since 2006, there has been an increase in the number of allo-HSCTs using alternative donors (Table 2).

**Source of hematopoietic stem cells **

Among the 32 auto-HSCTs, peripheral blood (PB) was the source of HSC in 25 (78.1%) patients, bone marrow (BM) in 1 (3.2%), and PB+BM in 6 (18.7%). Among the 222 allo-HSCTs, PB was the source of HSC in 159 (71.5%) patients, BM in 31 (14.0%), PB+BM in 1 (0.5%), BM+CB in 7 (3.1%), and CB in 24 (10.9%). The first allo-HSCT using CB was performed in 2005 and the number has increased every year since. 

**Outcome**

a. Survival 

At the time of analysis 172 of the patients were alive, 78 had died, and 4 had dropped out of the study. The 5-year EFS and OS was 55.7% ± 3.3% and 70.9% ± 3.3%, respectively. 

b. Mortality 

**Figure 2:**
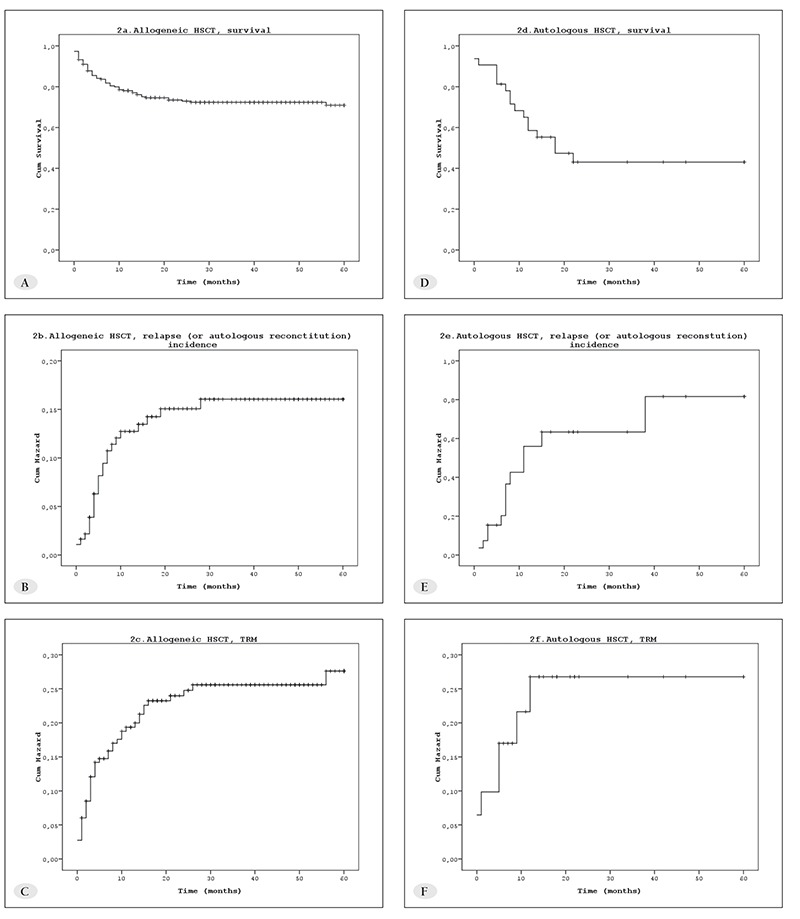
Main outcome of 254 children with HSCT (A: allogeneic HSCT, survival; B: allogeneic HSCT, relapse (or autologousreconstitution) incidence; C: allogeneic HSCT, TRM incidence; D: autologous HSCT, survival; E: autologous HSCT, relapse (orautologous reconstitution) incidence; F: autologous HSCT, TRM incidence)

The cause of death was transplant-related in 55 (70.6%) patients, and post-HSCT relapse or recurrence (for nonmalignant diseases) in 23 (29.4%) patients. The incidence of TRM, and relapse or recurrence differed between patients that underwent allo-HSCT and auto-HSCT. TRM, and relapse or recurrence at 5 years was 24.1% ± 3.3% and 14.8% ± 2.8% in allo-HSCT patients, respectively, and 23.5% ± 7.9%, and 55.8 ± 11.6% in auto-HSCT patients, respectively (Figure 2). The cumulative incidence of TRM in all the patients during 2 consecutive periods (1998-2003 and 2004-2008) did not differ significantly (24.2% ± 5.5% and 23.2% ± 3.3%, p<0.05, respectively).

**Figure 3:**
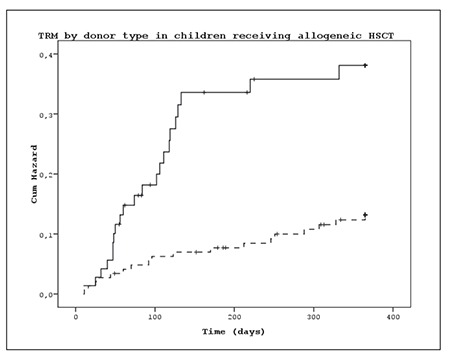
Cumulative incidence of TRM by donor type in children receiving allogeneic HSCT.

TRM post allo-HSCT differed according to donor type. Among the patients that underwent HSCT from an HLAidentical sibling donor, the cumulative incidence of TRM at 100 d and 365 d post HSCT was 5.4% ± 1.9% and 11.0% ± 2.6%, respectively, versus 16.6% ± 4.4% and 31.7% ± 5.6%, respectively, among those underwent allo- HSCT from an alternative donor (P<0.001) (Figure 3). TRM occured in 7 of the 32 patients that underwent auto- HSCT; 4 due to infection, of which 1 had graft failure, and other causes (veno-occlusive disease (VOD) [n = 2] and possible cardiac toxicity [n=1]). The primary diagnosis in the patients with TRM were as follows: neuroblastoma (n = 3), germ cell tumor (n=1), non-Hodgkin lymphoma (n=1), medulloblastoma (n = 1), and Ewing sarcoma (n = 1).

## DISCUSSION

This is the first study to evaluate and report on the development and recent activity in the field of HSCT in pediatric patients in Turkey. Analysis of the presented 10-year data set showed that HSCT is an accepted therapy, which is increasingly used. Auto-HSCT has an important role in a range of childhood cancers, such as neuroblastoma, lymphomas, and Ewing sarcoma/PNET [1,3]. In the present study the most common indications for auto-HSCT were neuroblastoma and lymphomas, which is consistent with other reports [2,3]. The reason for neuroblastoma was the consequence of clinical trials that reported auto-HSCT is more beneficial than conventional chemotherapy in children with high-risk neuroblastomas [4,5]. The Prospective Turkish Pediatric Oncology Group Neuroblastoma National Study included a high-dose chemotherapy and autologous hematopoietic stem cell rescue arm for highrisk neuroblastoma patients [6]. In this context, the number of transplants for neuroblastoma patients has been increasing since 2003 at our center, which is when the National Neuroblastoma Study began. 

Allogeneic transplantation is most frequently performed in children with high-risk and relapsed leukemias, myelodysplastic syndromes, aplastic anemia, congenital BM failure syndromes, beta thalassemia major, and various congenital metabolism disorders [1,7,8,9,10,11]. In the present study the most common indication for allo-HSCT was hemoglobinopathies, which was probably due to the high prevalence in our region [12] and referrals from other centers, followed by high-risk acute leukemias, including relapsed cases. During the study period, there was an increase in the number of allogeneic procedures using CB and stem cells from unrelated donors. CB has gradually emerged as an alternative source of hematopoietic cells for transplantation in children and adults with high-risk or advanced hematologic malignancies that do not have a suitably matched related or unrelated adult donor. This increase in the use of CB is due to favorable results in children, an increase in the availability of CB units with large cell doses, less stringent donor-recipient HLA matching, and rapid identification and acquisition of the unit. It was reported that DFS in children following CB transplantation is similar to that following allele-matched BM transplantation [13]. The primary obstacles to successful CB transplantation are delayed myeloid and thrombocyte engraftment time, and a higher rate of graft rejection than with BM- or PB-based HSCT [14].

Comparison of the present data on donors used in allo- HSCT with other similar international data showed that the number of transplantations from alternative donors is much higher in Australia than that in our center [15], whereas the number of transplantations from MSD at our center is similar to that in Switzerland and Eastern European countries [16, 17] and higher than that in Australia [15]. Despite the increase in the number of transplantations from the alternative donors at our center, this is not as high as that in developed counries, primarily because patients considered for transplantation do not present to our center in a timely fashion, the search for unrelated donors in Turkey cannot be completed in a timely fashion due to bureaucratic procedures, and the number of BM donors registered in Turkey has not reached the level in developed countries. 

In the present study the rate of TRM in the patients that underwent allo-HSCT was comparable to that reported in other studies [15,16,17]. In the present study’s allo-HSCT patients the primary causes of TRM were infection (43%) and GVHD (34%). Among the allo-HSCT patients, TRM during the first 5-years of the study did not differ from that during the second 5 years, during which time the number of transplantations from alternative donors substantially increased. The reason behind might be the decrease in TRM, at least, in transplantations from MSD based on experience obtained in the first 5 years. However, this was invalid for autologous transplantations, because the TRM rate in the auto-HSCT patients was much higher than acceptable thresholds. As fewer patients in the present study underwent auto-HSCT than allo-HSCT and the auto-HSCT patients were not homogeneous in respect to primary diagnosis, it is difficult associate this undesired outcome with a specific variable, such diagnosis, disease stage, or timing of transplantation. Among the patients with neuroblastoma, the most common indication for auto-HSCT (n=17), 3 patients died due to transplantrelated causes, of which 2 died due to severe VOD even thought they had not previously received abdominal irradiation and were in first remission. 

In conclusion, HSCT provided long-term survival and cured many of the patients with malignant and non-malignant disorders. The major cause of mortality was relapse of underlying conditions, which is indicative of the high-risk nature of diseases requiring HSCT. Despite the risks, we think HSCT offers the best chance of cure for many children with otherwise fatal diseases.

**Conflict of Interest Statement **

The authors of this paper have no conflicts of interest, including specific financial interests, relationships, and/ or affiliations relevant to the subject matter or materials included.

## Figures and Tables

**Table 1:**
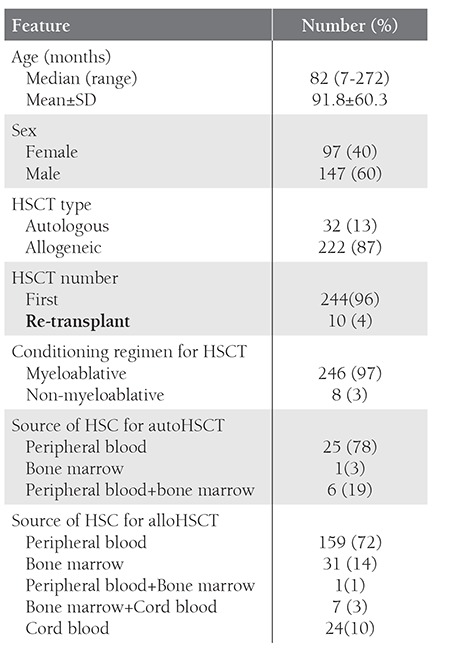
Characteristics of patients underwent HSCT during1998-2008.

**Table 2:**
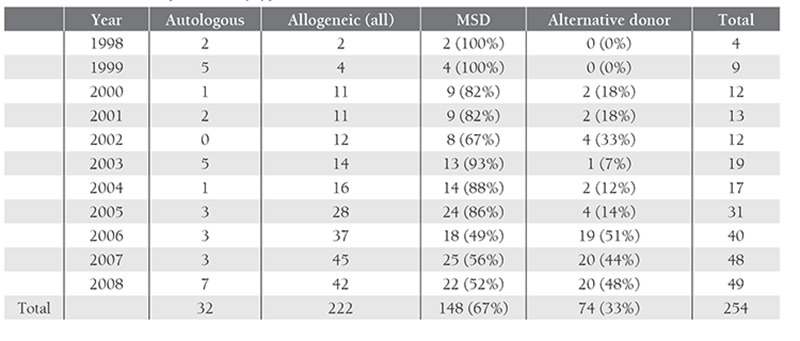
Number of HSCT performed, by type, 1998-2008 (n=254)
